# Ribosomal protein L32 enhances hepatocellular carcinoma progression

**DOI:** 10.1002/cam4.5811

**Published:** 2023-04-05

**Authors:** Guoxin Hou, Zhimin Lu, Jialu Jiang, Xinmei Yang

**Affiliations:** ^1^ Department of Oncology The First Hospital of Jiaxing, Affiliated Hospital of Jiaxing University Jiaxing Zhejiang China; ^2^ Department of outpatient The First Hospital of Jiaxing, Affiliated Hospital of Jiaxing University Jiaxing Zhejiang China

**Keywords:** bioinformatics, hepatocellular carcinoma, migration, RPL32, survival

## Abstract

**Purpose:**

The underlying mechanisms of hepatocellular carcinoma (HCC) have not been fully investigated, and effective biomarkers for HCC are still needed to be explored. Therefore, our study sought to thoroughly examine the clinical significance and biological functions of the ribosomal protein L32 (RPL32) in HCC by coupling bioinformatic methods with experimental analysis.

**Methods:**

To determine the clinical significance of *RPL32*, bioinformatic analyses were performed to examine *RPL32* expression in HCC patient samples and to correlate *RPL32* expression and HCC patient survival rates, genetic alterations, and immune cell infiltration. Cell counting kit‐8 assays, colony formation, flow cytometry, and transwell assays were performed to examine the effects of RPL32 on HCC cell proliferation, apoptosis, migration, and invasion in HCC cell lines (SMMC‐7721 and SK‐HEP‐1) where RPL32 was silenced using small interfering ribonucleic acid.

**Results:**

In the current study, we show that *RPL32* was highly expressed in HCC samples. Moreover, high levels of *RPL32* were associated with unfavorable outcomes in patients with HCC. Promoter methylation and copy number variation of *RPL32* were associated with *RPL32* mRNA expression. Results from the RPL32 silencing experiments indicated that the proliferation, apoptosis, migration, and invasion of SMMC‐7721 and SK‐HEP‐1 cells were attenuated upon RPL32 depletion.

**Conclusion:**

*RPL32* correlates with a favorable prognosis in patients with HCC and promotes the survival, migration, and invasion of HCC cells.

## Graphical abstract

Ribosomal protein L32 (RPL32) can be used as a prognostic biomarker for patients with hepatocellular carcinoma.

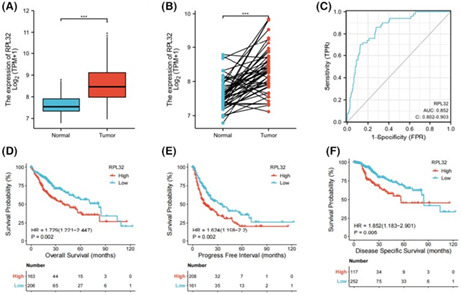

## 1 INTRODUCTION

Hepatocellular carcinoma (HCC) is the fourth leading cause of cancer‐related mortality worldwide.^1^ Despite a variety of effective therapeutic advances benefiting patients with HCC, the 5‐year survival of these patients is still low.^2^ HCC is thought to be induced by chronic liver disorders or hepatitis B infection.^3^ It has been suggested that pivotal genetic aberrations (i.e., mutations in *CTNNB1* and *P53* genes) may be triggered by these inducers of HCC tumorigenesis.^4^ Although targeted inhibitors for key genes, such as refametinib which blocks MEK activity,^5^ have been utilized clinically to intervene in HCC, patient survival improvement is still greatly hindered by drug resistance and cancer recurrence. Therefore, uncovering the mechanisms underlying HCC will contribute significantly to the identification of novel therapeutic targets, based on which adjuvant therapeutic strategies can be further explored. Furthermore, identifying effective prognostic biomarkers is important for the clinical treatment of patients with HCC.

Ribosomal protein (RP) L32 (RPL32), which encodes the 60S ribosomal subunit, is a cytoplasmic protein.^6^ RPs assemble ribosomes, and thus, play essential roles in protein translation. Although RPs function broadly in cellular processes, their expression patterns are unique in certain tissues, and some are dysregulated during pathological processes such as cancer.^7^ In specific cancers, including pancreatic and bladder cancer, specific RPs have been demonstrated to be misexpressed and enhance or inhibit cancer progression.^8^, ^9^, ^10^ In lung cancer, RPL32 was shown to interact with MDM2 protein (an E3 ligase for P53 protein), thereby enhancing the degradation of P53, resulting in lung cancer progression.^11^ *RPL32* expression was upregulated in breast cancer patient samples and cell lines. Functional investigation demonstrated that the loss of RPL32 leads to reduced breast cancer cell viability and migration.^12^ However, the expression pattern of *RPL32* in HCC patients and the biological functions of RPL32 during HCC progression have not been investigated.

In this study, we confirmed that *RPL32* expression was elevated in patients with HCC and was strongly associated with patient malignancy. Moreover, higher *RPL32* expression is associated with poor outcomes in patients with HCC. Importantly, RPL32 is an independent predictor of prognosis in HCC patients. In addition, we found that *RPL32* expression was strongly correlated with promoter methylation and copy number variation. The correlation between *RPL32* expression and infiltration of diverse immune cells was also systematically investigated. Finally, *RPL32* depletion significantly attenuated the viability, migration, and invasion of HCC cells. Our study couples bioinformatics methods and experimental analyses to thoroughly study the clinical significance and biological functions of RPL32 in HCC.

## 2 MATERIALS AND METHODS

### 2.1 Bioinformatic analysis

Raw RNA‐seq data of HCC patient samples and normal liver tissues were derived from TCGA‐LIHC (https://portal.gdc.cancer.gov/ gov/), GSE14520 (GPL3921 Subset),^13^ GSE76427,^14^ and ICGC‐LIRI‐JP^15^ datasets. Information on HCC patient survival and pathological parameters was downloaded from the TCGA‐LIHC, GSE14520, GSE76427, or ICGC‐LIRI‐JP databases. The promoter methylation and copy number variation (CNV) data were derived from the TCGA‐LIHC, LinkedOmics (http://www.linkedomics.org/)^16^ or UALCAN (http://ualcan.path.uab.edu/)^17^ databases. Normalization of all bioinformatics data was performed using the R “edgeR” package (version 3.30.3). |log2FC (fold change) | > 1 and FDR <0.05 were set as thresholds. The Kaplan–Meier curve R “survival” package (Version 3.1 12) was used to perform patient survival analyses. Immune cell infiltration and *RPL32* expression association were performed using the “GSVA” and “GSEABase” R packages, which were based on the ssGSEA algorithm.^18^ The R package “estimate” was used to check the correlations between gene expression and immune cell infiltration, based on the ESTIMATE algorithm.

### 2.2 Cell culture

HCC cell lines: L‐02, HCC‐LM3, Hep G2, Huh‐7, PLC/PRF/5, MHCC97‐H, SNU‐182, SMMC‐7721, and SK‐HEP‐1 cells (IMMOCELL, Xiamen), were maintained in Dulbecco's modified Eagle's medium (DMEM; Thermo Fisher, 41,966,029) supplemented with 10% fetal bovine serum (FBS; Thermo Fisher, 26,140,079), 100 U mL^−1^ penicillin, and 100 mg mL^−1^ streptomycin (PAN Biotech). Both cell lines were maintained in a humidified 5% CO_2_ atmosphere at 37°C.

### 2.3 Reverse transcription real‐time quantitative PCR (RT‐qPCR)

Total RNA was extracted using the TRIzol reagent (Invitrogen, 15596026) according to a standard protocol. Next, 1 μg of the isolated RNA was subjected to reverse transcription using the RevertAid First Strand cDNA Synthesis Kit (Thermo Fisher, K1621). The resulting cDNA served as a template for qPCR using the SYBR Select Master Mix (Thermo Fisher, 4309155). The primers used were *RPL32* F:5′‐ TCAAAATTAAGCGTAACTG‐3′, R:5’‐CTTCCATAACCAATGTTG‐3′, and *18S* F:5′‐ AGGCGCGCAAATTACCCAATCC‐3′, R:5’‐GCCCTCCAATTGTTCCTCGTTAAG‐3′. Signals were captured using a CFX Connect detection system (Bio‐Rad). The relative expression of *RPL32* RNA was calculated using the 2^−ΔΔCt^ algorithm. *18S* rRNA expression was used as an internal control for normalization.

### 2.4 Western Blotting

Cells were lysed in freshly prepared RIPA buffer (Beyotime, P0013B) with 1 × complete protease inhibitor cocktail on ice, and the protein concentration was determined using a bicinchoninic acid protein assay kit (Thermo Fisher, 23,225). Sodium dodecyl sulfate‐polyacrylamide gel electrophoresis (SDS‐PAGE) was performed to separate the proteins. The resolved proteins were transferred onto a 0.45‐μm polyvinylidene difluoride (PVDF) membrane. A blocking buffer consisting of 5% non‐fat dry milk in Tris‐buffered saline with 0.1% Tween 20 (TBST) was applied to the membranes to inhibit non‐specific binding. After 1 h incubation at room temperature, the blocked membranes were subjected to corresponding primary antibodies raised against RPL32 (Sabbiotech, 27,860–2) or β‐actin (Proteintech, Wuhan, China, 20,536‐1‐AP) diluted in TBST supplemented with 5% bovine serum albumin overnight at 4°C. The membranes were then washed with TBST three times before incubation with horseradish peroxidase (HRP)‐conjugated secondary antibody raised against mouse IgG (Proteintech, SA00001‐1) or rabbit IgG (Proteintech, SA00001‐2) for 2 h at room temperature. Finally, the signal was detected with the aid of Clarity™ Western ECL Substrate (Thermo Fisher, 32,209) and ChemiDoc Imaging System following the manufacturer's instructions.

### 2.5 Cell survival assay

SMMC‐7721 and SK‐HEP‐1 HCC cells were transfected with small interfering ribonucleic acid (siRNA) against *RPL32* (siRPL32, RiboBio) or non‐targeting siRNA using Lipofectamine 3000 (Thermo Fisher, L3000008). After 48 h of transfection, 10 μL/per well counting Kit‐8 (CCK‐8; Beyotime, C0037) reagent was added to the cells. After 1 h incubation at 37°C, the absorbance of cells at OD 450 nm was immediately determined using a microplate reader (Bio‐Rad).

### 2.6 Colony formation assay

Cells (1.5 × 10^3^) were seeded in a 6‐well plate and cultured for 14 days. The cells were washed with PBS, fixed in methanol for 15 min, and stained with crystal violet for 15 min. The colonies formed were then photographed and counted for statistical analysis.

### 2.7 Cell cycle analysis

Cell cycle analysis was performed using flow cytometry. Briefly, cells were dissociated into single‐cell suspensions and stained with propidium iodide (PI; Vazyme; A211‐02). Flow cytometry was performed using a NovoCyte (ACEA), and the data were analyzed using NovoExpress 1.4.1.

### 2.8 Cell apoptosis analysis

The Annexin V‐FITC Kit (Beyotime Biotechnology) was used to measure the apoptosis of SMMC‐7721 and SK‐HEP‐1 cells treated with siRPL32. After treatment and isolation, cells were stained with PI staining solution and Annexin V‐FITC at 25°C for 15 min away from light according to the manufacturer's instructions. Subsequently, the apoptosis rate was analyzed using flow cytometry and associated software.

### 2.9 Cell migration and invasion assays

5 × 10^5^ SMMC 7721 cells were plated in the top chamber of an 8 μm‐pore 24‐well transwell plate (Corning, 3422) in DMEM without serum. The bottom chamber was filled with DMEM containing 10% FBS. After 2 days of culture, the cells on the bottom of the membrane were fixed, stained, and photographed. For invasion analysis, the membrane was precoated with Matrigel (BD Biosciences).

### 2.10 Statistical analyses

GraphPad Prism version 9.0 software was used for statistical analyses and all the quantitative data are shown as mean ± standard deviation. Statistical significances were calculated by an unpaired Student's *t*‐test and *p* < 0.05 was considered as statistically significant (*0.01 < *p* < 0.05, **0.001 < *p* < 0.01, ***0.0001 < *p* < 0.001). ns, not significant.

## 3 RESULTS

### 3.1 *RPL32* expression is elevated in HCC patients

**FIGURE 1:**
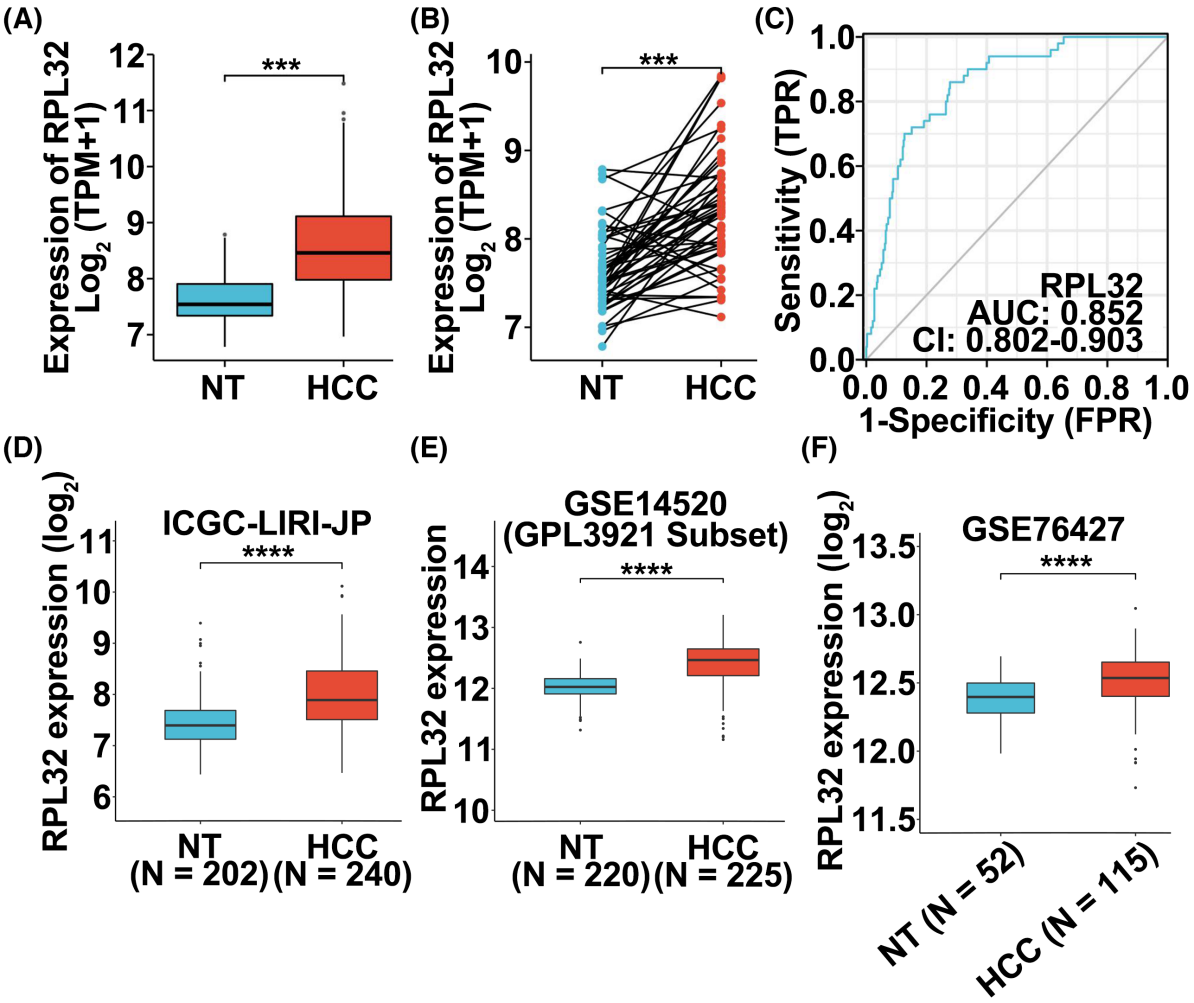
*RPL32* expression is elevated in HCC patients. (A, B) Unpaired (A) and paired (B) differential expression analysis of *RPL32* between normal and tumor tissue samples from the TCGA‐LIHC database. (C) Estimated ROC curve to measure the diagnostic value (by analyzing sensitively and specificity) of *RPL32* in the TCGA‐LIHC database. (D–F) Unpaired differential expression analysis of *RPL32* between non‐tumor (NT) and HCC tissue samples from the GSE14520 (D), ICGC‐LIRI‐JP (E), and GSE76427 (F) datasets.

To evaluate the expression pattern of *RPL32* in a pan‐cancer manner, we performed bioinformatics analysis on TNMplot, TIMER, and GTEx (coupled with TCGA) databases, and found that *RPL32* was highly expressed in a large proportion of cancers (Figure S1A–C), indicating *RPL32* is an oncogene. We focused on HCC due to the lack of *RPL32* investigation in HCC. Baseline demographic, clinicopathologic, and tumor characteristics of patients with HCC from TCGA‐LIHC are summarized in Table 1. Surprisingly, a dramatic increase in *RPL32* expression levels was found in HCC samples compared to normal tissue samples (through both unpaired and paired comparisons) in the TCGA‐LIHC database (Figure 1A,B). Importantly, the estimated receiver operator characteristic (ROC) curve revealed the diagnostic sensitivity and specificity of *RPL32* in patients with HCC (area under the ROC curve [AUC] = 0.852; Figure 1C). In addition, data from ICGC‐LIRI‐JP, TNMplot, UALCAN (TCGA), and gene expression omnibus (accession numbers: GSE14520, GSE76427, GSE14811, GSE36376, GSE112790) showed that *RPL32* expression levels were higher in HCC tissues than in normal tissues (Figure 1D–F, Figure S1D–I). These findings strongly suggested that RPL32 may contribute to HCC tumorigenesis. Moreover, the correlations between *RPL32* expression and clinical stages were explored using the TCGA‐LIHC database, in which such information is accessible. Although *RPL32* was not differentially expressed in patients classified into different T stages, N stages, M stages, pathologic stages, Child–Pugh grades, adjacent hepatic tissue inflammation levels, or vascular invasion levels (Figure S2A–G), *RPL32* mRNA was positively correlated with higher histologic grade and higher alpha fetoprotein (AFP) levels (Figure S2H–I). These results suggest that *RPL32* expression was enhanced in patients with HCC.

**TABLE 1:** Demographic, clinical, and histopathologic characteristics of patients with HCC from TCGA‐LIHC (*n* = 371).

Characteristic	Levels	Number (%)
T stage	T1	181 (49.2%)
	T2	94 (25.5%)
	T3	80 (21.7%)
	T4	13 (3.5%)
N stage	N0	252 (98.4%)
	N1	4 (1.6%)
M stage	M0	266 (98.5%)
	M1	4 (1.5%)
Pathologic stage	Stage I	171 (49.3%)
	Stage II	86 (24.8%)
	Stage III	85 (24.5%)
	Stage IV	5 (1.4%)
Tumor status	Tumor free	201 (57.1%)
	With tumor	151 (42.9%)
Gender	Female	121 (32.6%)
	Male	250 (67.4%)
Race	Asian	158 (44%)
	Black or African American	17 (4.7%)
	White	184 (51.3%)
Age	≤60	177 (47.8%)
	>60	193 (52.2%)
Weight	≤70	182 (52.9%)
	>70	162 (47.1%)
Height	<170	199 (58.7%)
	≥170	140 (41.3%)
BMI	≤25	177 (52.8%)
	>25	158 (47.2%)
Histologic grade	G1	55 (15%)
	G2	177 (48.4%)
	G3	122 (33.3%)
	G4	12 (3.3%)
Adjacent hepatic tissue inflammation	None	117 (50%)
	Mild	99 (42.3%)
	Severe	18 (7.7%)
AFP (ng/mL)	≤400	213 (76.6%)
	>400	65 (23.4%)
Prothrombin time	≤4	206 (70.1%)
	>4	88 (29.9%)
Child–Pugh grade	A	217 (90.8%)
	B	21 (8.8%)
	C	1 (0.4%)
Vascular invasion	No	206 (65.4%)
	Yes	109 (34.6%)

### 3.2 High *RPL32* expression is correlated with shorter survival in HCC patients

**FIGURE 2:**
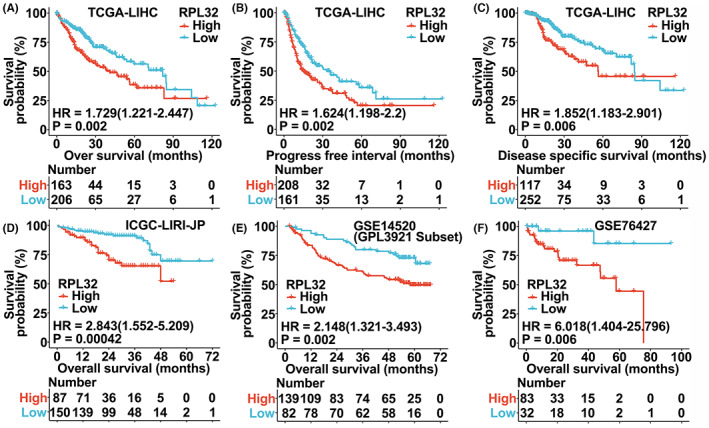
*RPL32* is correlated with the unfavorable prognosis in patients with HCC. (A–C) Kaplan–Meier analysis for HCC patients stratified by *RPL32* expression in the TCGA‐LIHC database. Overall survival (A), progression‐free interval (B), and disease‐specific survival (C) were quantified. (D–F) Overall survival of HCC patients divided by *RPL32* levels in GSE14520 (D), ICGC‐LIRI‐JP (E), or GSE76427 (F) databases.

Next, we determined the correlation between *RPL32* expression and HCC patient survival. Notably, HCC patients with higher *RPL32* expression levels displayed worse prognosis in terms of overall survival, progression‐free survival, and disease‐specific survival in multiple databases (Figure 2A–F). In addition, Kaplan–Meier data from the UALCAN database also revealed that *RPL32* expression was negatively correlated with patient overall survival and relapse‐free interval (Figure S3A–I). More specifically, we were interested in whether *RPL32* expression was associated with patient survival in certain subtypes of HCC. Therefore, we assessed the survival of patients with HCC in various subgroups. Importantly, we observed that in multiple subgroups, including T stage (T1, T3), pathologic stage (I, III), histologic grade (G1, G2, G3, and G4), AFP ≤400, and adjacent hepatic tissue inflammation (none, mild, and severe), HCC patients with higher *RPL32* expression exhibited lower survival probabilities (Figure S4A–I). In summary, *RPL32* expression was significantly correlated with unfavorable outcomes in patients with HCC with different clinical characteristics, which confirms the general use of *RPL32* expression as a prognostic biomarker.

### 3.3 *RPL32* expression is an independent parameter for HCC patient prognosis

**FIGURE 3:**
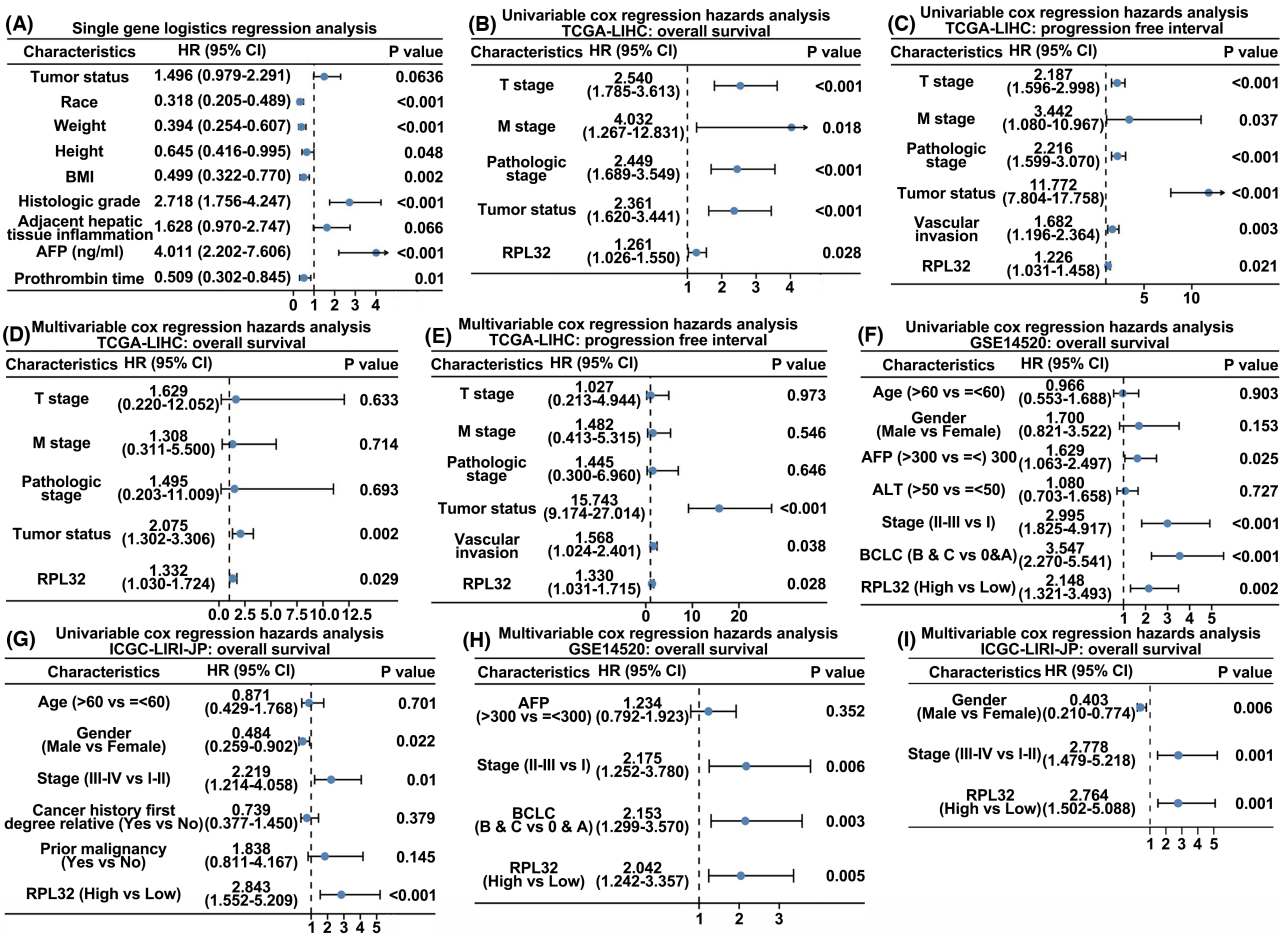
Cox regression hazard analysis of survival for HCC cancer patients. (A) Single‐gene logistics regression analysis (TCGA‐LIHC database). (B) Univariable Cox regression hazards analysis of clinical parameters and *RPL32* expression regarding overall survival (TCGA‐LIHC database). (C) Univariable Cox regression hazards analysis of clinical parameters and *RPL32* levels regarding progression‐free survival (TCGA‐LIHC database). (D) Multivariable Cox regression hazards analysis of clinical parameters and *RPL32* expression regarding overall survival (TCGA‐LIHC database). (E) Multivariable Cox regression hazards analysis of clinical parameters and *RPL32* regarding progression‐free survival (TCGA‐LIHC database). (F) Univariable Cox regression hazards analysis of clinical parameters and RPL32 expression regarding overall survival (GSE14520). (G) Univariable Cox regression hazards analysis of clinical parameters and RPL32 levels regarding overall survival (ICGC‐LIRI‐JP). (H) Multivariable Cox regression hazards analysis of clinical parameters and RPL32 expression regarding overall survival (GSE14520). (I) Multivariable Cox regression hazards analysis of clinical parameters and RPL32 levels regarding overall survival (ICGC‐LIRI‐JP).

Given the fact that clinical classification standards, such as T stage, M stage, and tumor status, are vital parameters for patient outcome prediction, we continued the clinical relevance analysis by determining whether *RPL32* expression could serve as an independent factor for predicting patient malignancy. First, single‐gene logistic regression analysis was performed to determine which clinical parameters were significantly correlated with the patient malignancy classification (Figure 3A). Moreover, multivariate and univariate Cox analyses of these clinical factors or *RPL32* expression levels demonstrated that *RPL32* expression was a potential predictor of HCC patient prognosis (Figure 3B–E). This conclusion was further validated in HCC cohorts from the GSE14520 and ICGC‐LIRI‐JP databases (Figure 3F–I). The baseline demographic, clinicopathologic, and tumor characteristics of patients with HCC from GSE14520 and ICGC‐LIRI‐JP are summarized in Tables 2 and 3. Furthermore, to better quantify the risk and predict the survival of patients with HCC, a nomogram combining *RPL32* expression with other effective clinical parameters was established (Figure S5A). Notably, positive correlations between the predicted and actual survival probabilities were observed in the diverse patient cohorts (Figure S5B–D). Collectively, these results suggest that *RPL32* may act as an independent and effective predictor of patient survival.

**TABLE 2:** Demographic, clinical, and histopathologic characteristics of patients with HCC from GSE14520 (*n* = 221).

Characteristic	Levels	Number (%)
Pathologic stage	Stage I	93 (42.1%)
	Stage II	77 (34.8%)
	Stage III	49 (22.2%)
	Unknown	2 (0.9%)
Gender	Female	30 (13.6%)
	Male	191 (86.4%)
Age	≤60	181 (81.9%)
	>60	40 (18.1%)
AFP	≤300	118 (53.4%)
	>300	100 (45.2%)
	Unknown	3 (1.4%)
ALT	≤50	130 (58.8%)
	>50	91 (41.2%)
BCLC	0	20 (9.0%)
	A	148 (67.0%)
	B	22 (10.0%)
	C	29 (13.1%)
	N/A	2 (0.9%)

**TABLE 3:** Demographic, clinical, and histopathologic characteristics of patients with HCC from ICGC‐LIRI‐JP (*n* = 237).

Characteristic	Levels	Number (%)
Pathologic stage	Stage I	35 (14.8%)
	Stage II	107 (45.1%)
	Stage III	74 (31.2%)
	Stage IV	21 (8.9%)
Gender	Female	60 (25.3%)
	Male	177 (74.7%)
Age	≤60	49 (20.7%)
	>60	188 (79.3%)
Cancer history first‐degree relative	No	146 (61.6%)
	Unknown	15 (6.3%)
	Yes	76 (32.1%)
Status	Alive	194 (81.9%)
	>4	43 (18.1%)
Prior malignancy	No	207 (87.3%)
	Yes	30 (12.7%)

### 3.4 Promoter methylation and copy number variation affect *RPL32* mRNA levels in HCC patients

**FIGURE 4:**
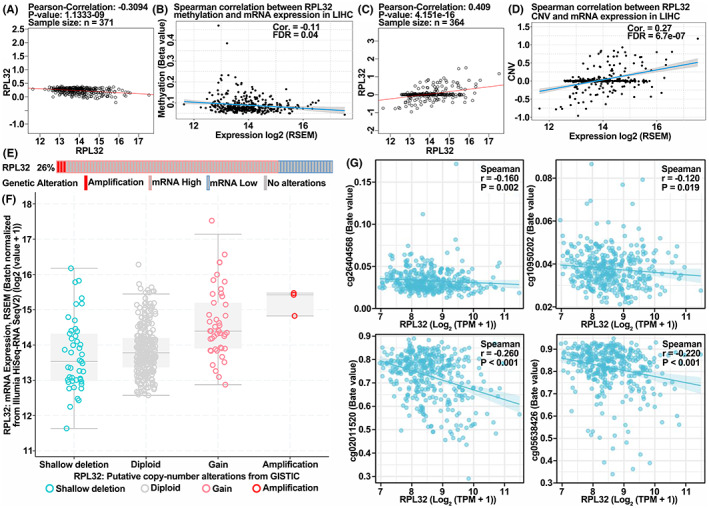
*RPL32* mRNA expression is correlated with its promoter methylation and copy number variation. (A, B) Scatter plots illustrating the correlations between *RPL32* mRNA levels and the methylation of promoters in HCC patients from the LinkedOmics database (A) and TCGA‐LIHC database (B). (C, D) Scatter plots suggesting the correlations between *RPL32* mRNA levels and the copy number variations (CNVs) in HCC patients from the LinkedOmics database (C) and TCGA‐LIHC database (D). (E) Schematic plot showing the proportion and types of the genetic alterations of *RPL32*. (F) Correlation between genetic alterations and the mRNA expression of *RPL32* in HCC patients. (G) Correlation between *RPL32* mRNA expression and the methylation levels of ERCC6L promoter at the four indicated methylation points in HCC patients.

As *RPL32* expression was shown to be dysregulated during HCC progression, we next analyzed whether promoter methylation and CNV of *RPL32* expression were altered in patients with HCC. Analysis of data from LinkedOmics and TCGA‐LIHC databases revealed negative correlations between *RPL32* mRNA levels and promoter methylation (Figure 4A, B). Interestingly, CNV was positively associated with *RPL32* mRNA expression (Figure 4C, D). Moreover, genetic alterations were frequently detected in HCC patients (26%; Figure 4E, F). In addition, we found an inverse correlation between *RPL32* expression and methylation at four methylation sites, cg26404568, cg10950202, cg2011520, and cg5638426, in the promoter region (Figure 4G). Taken together, these results reveal that *RPL32* mRNA level variation attributes to promoter methylation and copy number alterations in patients with HCC.

### 3.5 Correlation between *RPL32* expression and immune cell infiltration

**FIGURE 5:**
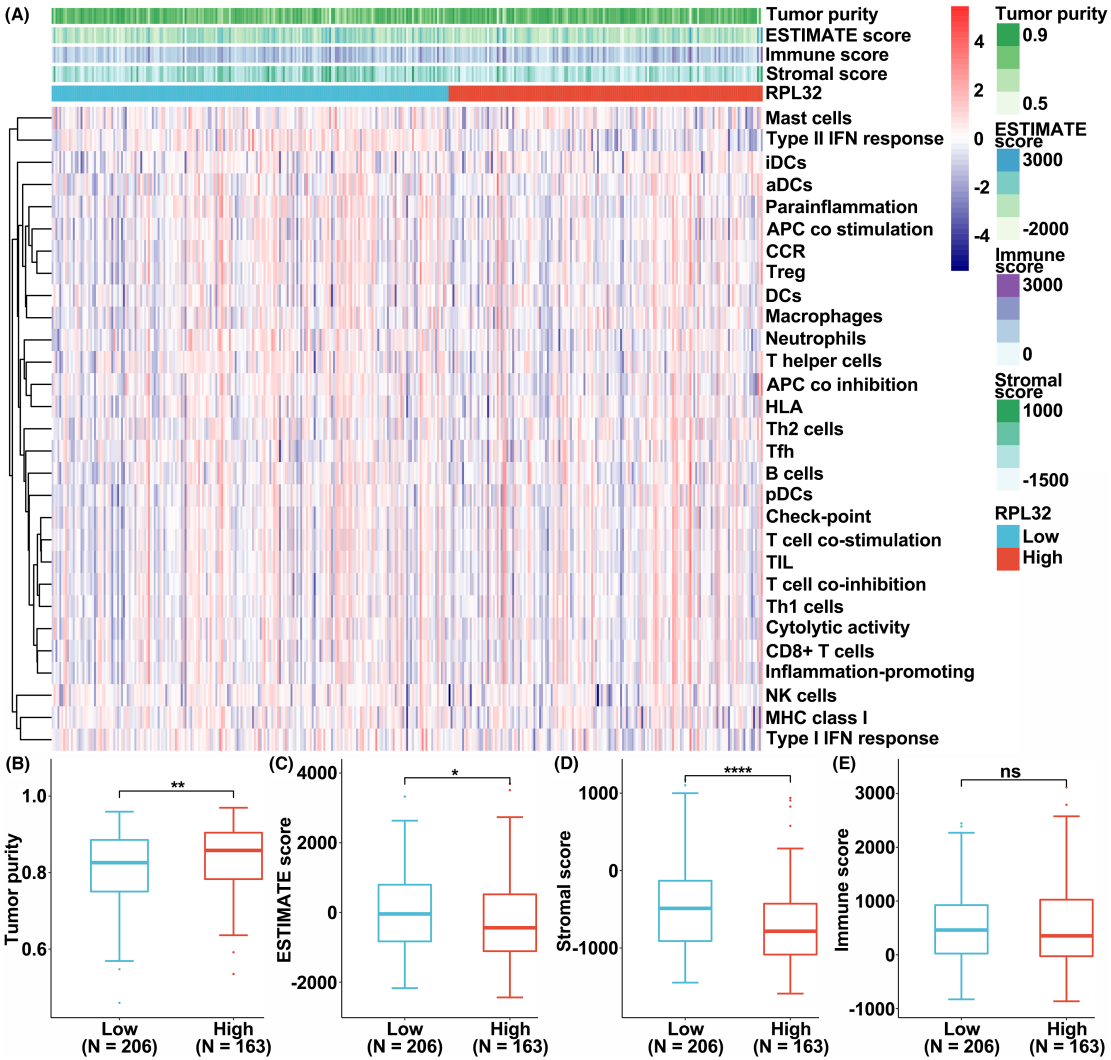
Correlation of RPL32 level with immune and stromal scores in HCC. (A) Heatmap showing the levels of *RPL32* mRNA (clustered as low and high) and infiltration of various immune cells. The infiltration of immune cells was calculated by the ssGSEA algorithm. (B–E) Differential comparison of the Tumor Purity (B), ESTIMATE Score (C), Stromal score (D), or Immune Score (E) in HCC patients stratified by *RPL32* mRNA levels (low, *N* = 206; high, *N* = 163).

Cancer progression is related to the type of immune infiltrating cells^19^; therefore, we determined the correlations between *RPL32* expression and the infiltration of immune cells on the GSCA platform.^20^ We determined that RPL32 levels were associated with immune cell infiltration in HCC, based on the combined scores (Figure 5A). Patients with higher *RPL32* expression exhibited higher tumor purity and lower ESTIMATE scores as well as stromal scores, with no difference in terms of the immune score (Figure 5B–E). Furthermore, we found that *RPL32* expression was positively associated with the infiltration of several immune cells, such as CD8 T cells, gamma delta T cells, effector memory T cells, and B cells (Figure S6A–E). Nevertheless, inverse correlations were observed in central memory cells, iTreg cells, CD4 naïve T cells, and Th17 cells (Figure S6A, F–I). We also quantified patient survival in subgroups with specific immune cell enrichment. As shown in Supplementary Figures S7 and S8, negative correlations between *RPL32* levels and overall survival of patients with HCC were detected in all the indicated subclasses of patients. In addition, *RPL32* expression was negatively associated with resistance to several drugs such as 5‐Fluorouracil, I‐BET‐762, and BHG712 (Figure S9).

### 3.6 Loss of RPL32 mitigates the survival, migration, and invasion of HCC cells

**FIGURE 6:**
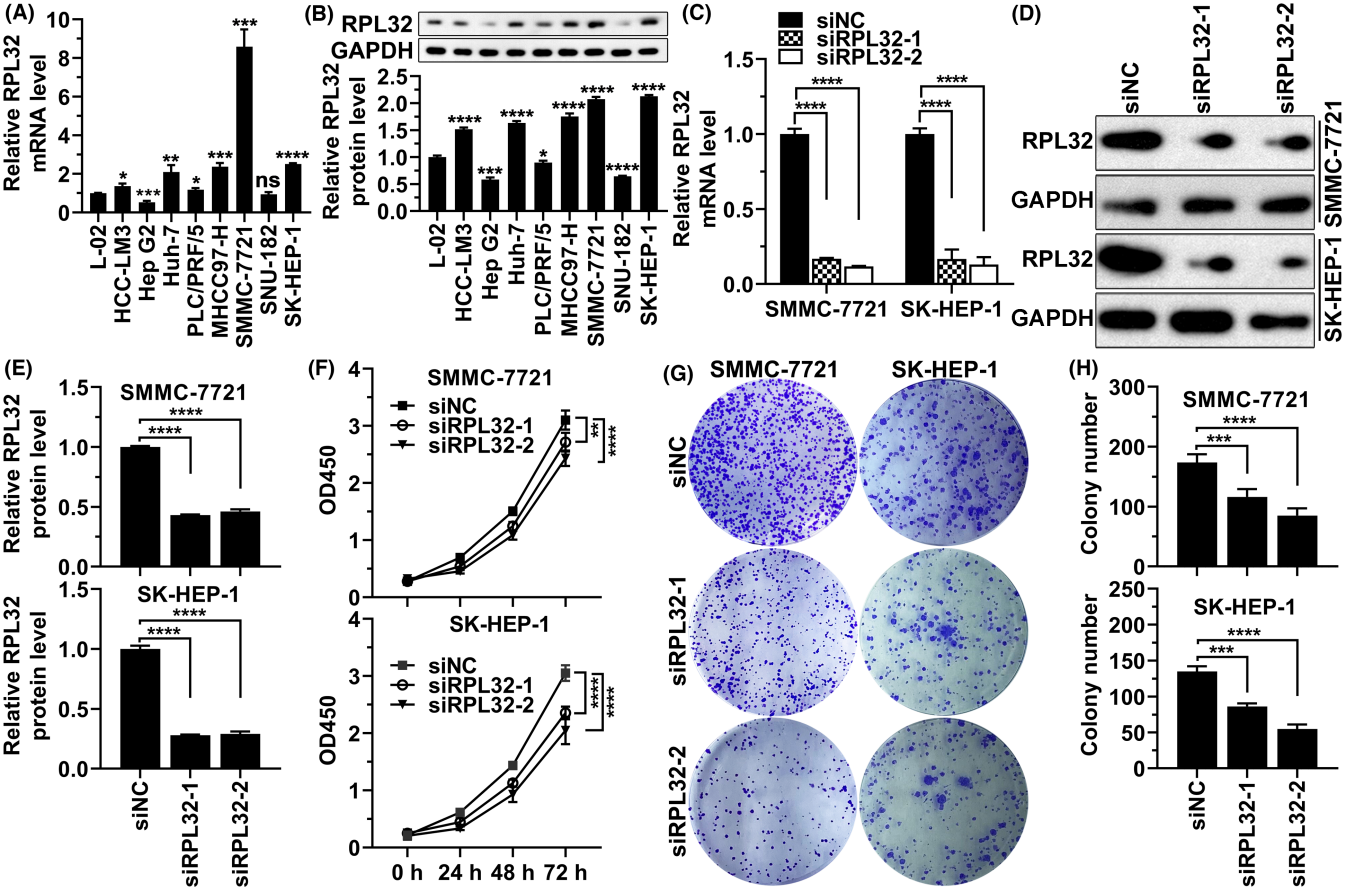
RPL32 silencing mitigates the survival and colony formation of HCC cells. (A) RT‐qPCR detection of *RPL32* mRNA expression in L‐02, HCC‐LM3, Hep G2, Huh‐7, PLC/PRF/5, MHCC97‐H, SMCC‐7721, SNU‐182, and SK‐HEP‐1 HCC cells. (B) Representative western blotting images (top) and their quantification (bottom) of RPL32 protein levels in L‐02, HCC‐LM3, Hep G2, Huh‐7, PLC/PRF/5, MHCC97‐H, SMCC‐7721, SNU‐182, and SK‐HEP‐1 cells. (C) RT‐qPCR detection of *RPL32* mRNA expression after siRNA transfection in SMCC‐7721 and SK‐HEP‐1 cells. (D, E) Representative western blotting images (D) and their quantification (E) of RPL32 protein levels after siRNA transfection in SMCC‐7721 and SK‐HEP‐1 cells. (F) CCK‐8 assay values as measured at OD450 demonstrating cell survival of SMCC‐7721 and SK‐HEP‐1 cells after siRNA transfection. (G and H) Representative images (G) and quantification results (H) from colony formation assays of SMMC7721 and SK‐HEP‐1 cells after transfecting with two siRNAs against *RPL32*.

**FIGURE 7:**
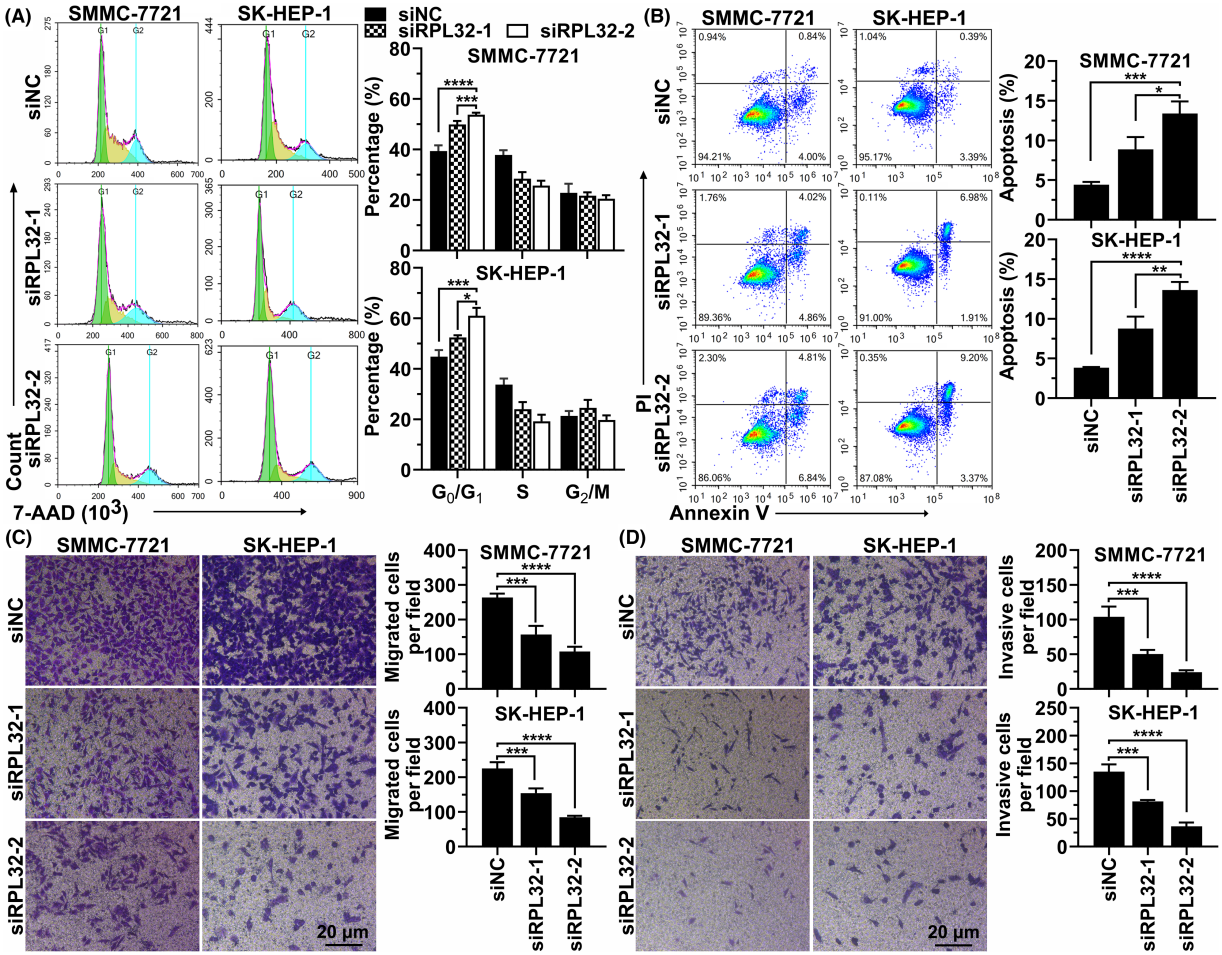
RPL32 silencing mitigates cell cycle progression, migration, and invasion and enhances apoptosis in HCC cells. (A). Flow cytometry analysis (left) and quantification (right) evaluating the cell cycle proportions of SMMC7721 and SK‐HEP‐1 cells transfected with two separate siRNAs against *RPL32*. (B) Flow cytometry analysis (left) and quantification (right) evaluating the apoptosis of SMMC7721 and SK‐HEP‐1 cells transfected with two separate siRNAs against *RPL32*. (C and D) Representative images and quantification results from transwell assays for assessing the migration (C) and invasion (D) of SMMC7721 and SK‐HEP‐1 cells after transfection with two siRNAs against *RPL32*.

To further uncover the biological effects of RPL32 in HCC cells, we detected the mRNA and protein levels of RPL32 in different HCC cell lines and found that, except for Hep G2, PLC/PRF/5, and SNU‐182 cell lines, the expression level of RPL32 in most HCC cell lines (HCC‐LM3, Huh‐7, MHCC97‐H, SMMC‐7721, and SK‐HEP‐1) was significantly higher than that in normal liver cells (L‐02) (Figure 6A, B). Because the expression level of RPL32 was the highest in SMMC‐7721 and SK‐HEP‐1 cells (Figure 6A, B), *RPL32* was knocked down by two independent siRNAs in the HCC cell lines SMMC‐7721 and SK‐HEP‐1. Successful knockdown of *RPL32* at the mRNA and protein levels was detected using RT‐qPCR and western blotting, respectively (Figure 6C–E). Next, we evaluated the effect of *RPL32* expression on cell survival. As determined by CCK‐8 and colony formation analyses, HCC (SMMC‐7721 and SK‐HEP‐1) cell survival and colony formation were greatly diminished upon RPL32 depletion (Figure 6F–H). Importantly, flow cytometry assays revealed that cell cycle progression was hindered when RPL32 was silenced in HCC cells (Figure 7A). However, decreased expression of RPL32 enhanced apoptosis (Figure 7B). Furthermore, the absence of RPL32 resulted in attenuated HCC cell migration and invasion (Figure 7C, D). Overall, these data demonstrated that RPL32 knockdown alleviates the survival, migration, and invasion of HCC cells.

## 4 DISCUSSION

Increasing evidence shows that there are about 80 kinds of ribosomal proteins in eukaryotes, which not only function to form ribosomes and bind RNA but also have other functions, such as participating in tumor development and regulating tumor resistance to drugs.^11^, ^21^ Today, many ribosomal proteins have been found to be involved in regulating the occurrence and development of a variety of cancers, including lung, breast, and liver cancers.^11^, ^12^, ^22^ In addition, RNA‐binding protein (RBP), a key regulator of gene expression, is involved in the progression of many cancers, such as HCC.^22^, ^23^, ^24^ A total of 526 RBPs were abnormally expressed in HCC compared to normal adjacent liver tissues and predicted poor prognosis.^25^ A study showed that abnormal expression of RBPs in HCC enhanced tumor metastasis and accelerated tumor progression.^26^ Currently, about 1542 human RBPs have been found to be involved in cancer development and progression.^27^ However, the number of well‐characterized RBPs in HCC remains unknown. RPL32 is a ribosomal component protein^28^ and RNA‐binding protein.^29^ In this study, abnormal expression of RPL32 in HCC was found to be closely related to tumor progression.

In vitro experiments showed that RPL32 depletion had no effect on the proliferation of breast cancer cells^12^ but inhibited the proliferation of lung cancer cells.^11^ These findings suggest that RPL32 plays different roles in the proliferation of different cancer cells. In our study, the knockdown of RPL32 significantly reduced the proliferation of SMMC‐7721 and SK‐HEP‐1 cells. Additionally, silencing RPL32 blocks the cell cycle in lung cancer cells.^11^ Similarly, we found that RPL32 deficiency blocked the cell cycle progression of SMMC‐7721 and SK‐HEP‐1 cells in the G_0_/G_1_ phase. Moreover, in yeast, which are also eukaryotic cells, high expression of RPL32‐2 promotes cell proliferation, while increased expression of RPL32‐1 inhibits cell division.^21^ This may be one of the mechanisms by which RPL32 plays different roles in the proliferation of different cancer cells.

In chronic lymphocytic leukemia (CLL), *RPL32* expression can be used to predict *SF3B1* expression, the major recurrent mutant gene in CLL.^30^ The expression level of RPL32 in late androgen‐independent cells was significantly higher than that in early androgen‐sensitive cells, suggesting that RPL32 may be positively correlated with the progression of prostate cancer.^31^ In breast cancer patients, RPL32 expression is higher in circulating tumor cell clusters with greater metastatic potential than in single circulating tumor cells.^32^ In addition, silencing of RPL32 significantly reduces cell migration and invasion in breast cancer cells.^12^ These results suggest that RPL32 is closely related to tumor metastasis. Similarly, we found that silencing RPL32 resulted in a significant decrease in the number of cells that migrated and invaded. Furthermore, the reduction of RPL32 in SMMC‐7721 and SK‐HEP‐1 cells enhanced apoptosis. Although the increase in apoptosis and decrease in proliferation may also lead to a decrease in the number of migrating and invading cells to a certain extent, a prior study showed that the loss of RPL32 in breast cancer reduces the protein levels of matrix metalloproteinase (MMP)‐2 and MMP‐9, and significantly reduces cell migration and invasion,^12^ suggesting that RPL32 has the potential to inhibit the migration and invasion of cancer cells by downregulating the expression of MMPs.

One study examined whether RPL32 was associated with cisplatin sensitivity in the lung cancer cell lines A549, NCI‐H460, and H1299 and showed that knockout of RPL32 significantly increased cisplatin sensitivity in A549 and NCI‐H460 cells.^11^ In this study, we also analyzed the relationship between RPL32 and HCC drug sensitivity and found that RPL32 expression was negatively correlated with HCC sensitivity to many drugs, including BHG712, I‐BET‐762, and 5‐fluorouracil.

Genetic variation is a major cause of gene dysregulation in cancer.^33^ Promoter methylation at CpG sites leads to gene transcription silencing, which usually occurs in tumor suppressors and results in cancer progression.^34^ In addition, CNV, such as depletion and gain of copy numbers due to amplification, also contributes to a certain gene expression and is a frequent event in tumor‐related genes.^35^ Therefore, quantification of *RPL32* promoter methylation and CNV in HCC patients was carried out, and the correlations between them and *RPL32* mRNA expression were studied. As expected, *RPL32* mRNA levels were negatively and positively associated with its promoter methylation and CNV, respectively.

In brief, in the present study, we analyzed HCC patient data from different databases and systematically revealed that the expression of RPL32 in HCC samples was significantly higher than that in normal tissue samples, which was associated with a lower survival probability in HCC patients. Additionally, RPL32 can be used as an independent predictor of HCC. Moreover, in vitro experiments revealed that RPL32‐knockdown in SMMC‐7721 and SK‐HEP‐1 HCC cells reduced cell survival, migration, and invasion. These results suggest that RPL32 can be used as a prognostic biomarker and promising therapeutic target for patients with HCC.

## AUTHOR CONTRIBUTIONS

**Guoxin Hou:** Conceptualization (lead); data curation (lead); formal analysis (lead); funding acquisition (supporting); investigation (lead); methodology (lead); project administration (lead); writing – original draft (lead); writing – review and editing (lead). **Zhimin Lu:** Conceptualization (equal); data curation (equal); formal analysis (equal); investigation (equal); methodology (equal). **Jialu Jiang:** Data curation (equal); formal analysis (equal); investigation (equal). **Xinmei Yang:** Funding acquisition (lead); supervision (lead); validation (lead); visualization (lead); writing – review and editing (equal).

## FUNDING INFORMATION

This research was supported by the Zhejiang Provincial Natural Science Foundation of China under grant no. LY20H160041, Medicine and Health Technology Plan Project of Zhejiang Province (2022KY371), Jiaxing Key Laboratory of Oncology radiotherapy (2021‐zlzdsys), 2023 Jiaxing Key Discipline of Nursing (Supporting Subject) (2023‐ZC‐007), and 2019 Jiaxing Key Discipline of Medicine‐Oncology (Supporting Subject) (2019‐zc‐11).

## CONFLICT OF INTEREST STATEMENT

The authors declare that they have no competing interests.

## ETHICS APPROVAL AND CONSENT TO PARTICIPATE

Not applicable.

## Supporting information


**Data S1:** Supporting Information

## Data Availability

Data sharing is not applicable to this article as no new data were created or analyzed in this study.
