# Supplementary material for: Ribosomal protein L32 enhances hepatocellular carcinoma progression
Source: Cancer Med. 2023 Apr 5;12(9):10791–803. doi: 10.1002/cam4.5811 (PMC10225200; doi:10.1002/cam4.5811)
Supplement: Supplementary file 1 — Data S1: Supporting Information [file CAM4-12-10791-s001.pdf]

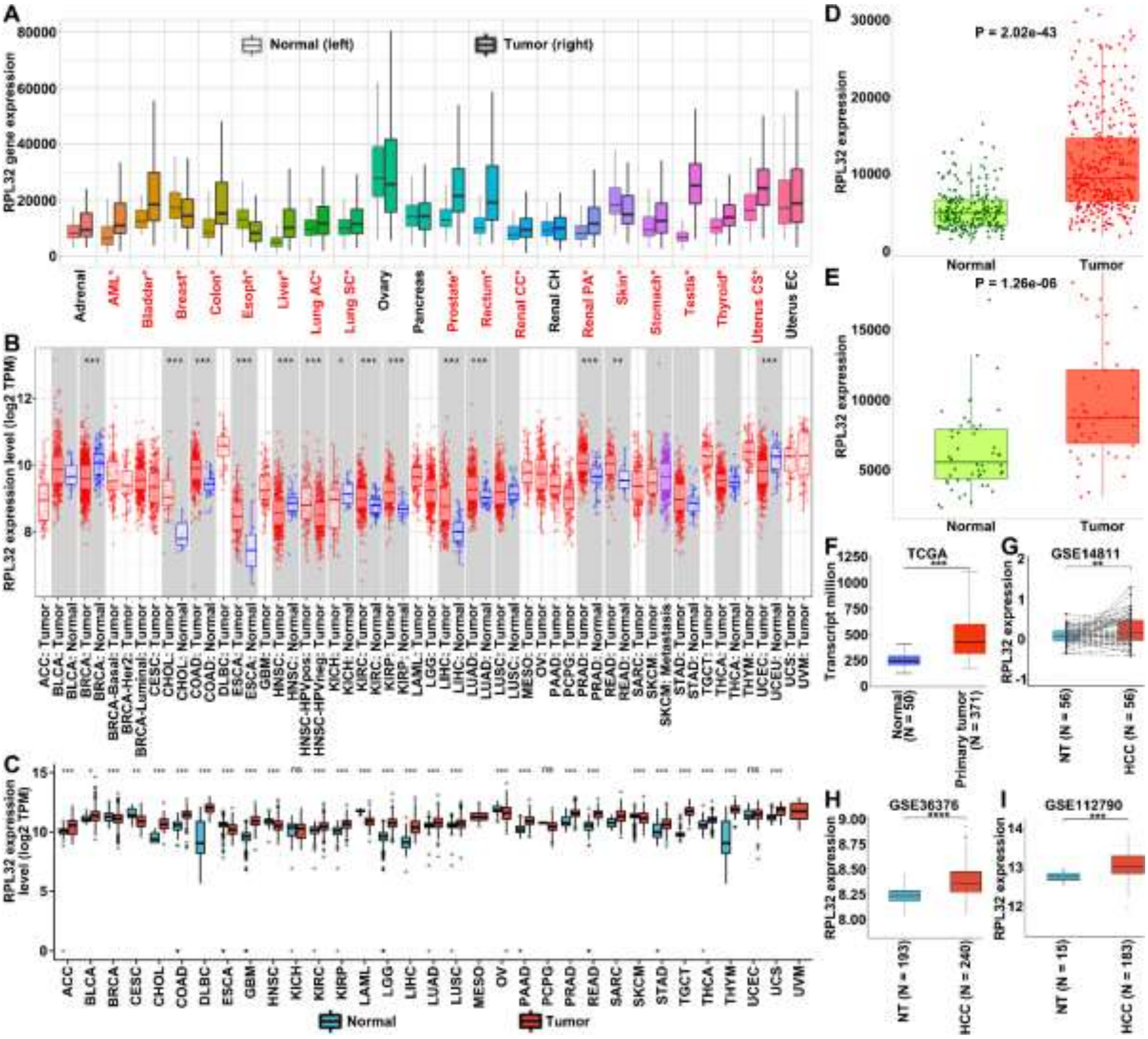

Figure S1. Pan-cancer analysis of *RPL32* expression

(A) Plot showing the expression of *RPL32* in normal (left) or tumor (right) samples from multiple cancer types. The figure was generated based on the transcriptomic data from the TNMplot database (https://tnmplot.com/analysis/). (B, C) Plot indicating *RPL32* levels in normal and tumor samples. The figure was generated on the transcriptomic data from the TIMER database (B, https://cistrome.shinyapps.io/timer/) and GTEx coupled with TCGA (C), respectively. (D-F) Differential expression analysis of *RPL32* between normal and HCC tumor samples in the TNMplot database including all the samples (D) or only paired samples (E), in the UALCAN database (http://ualcan.path.uab.edu/; F). (G-I) Differential expression analysis of *RPL32* between non-tumor (NT) and HCC tissue samples from the GSE14811 (G), GSE36376 (H) and GSE112790 (I) dataset, respectively.

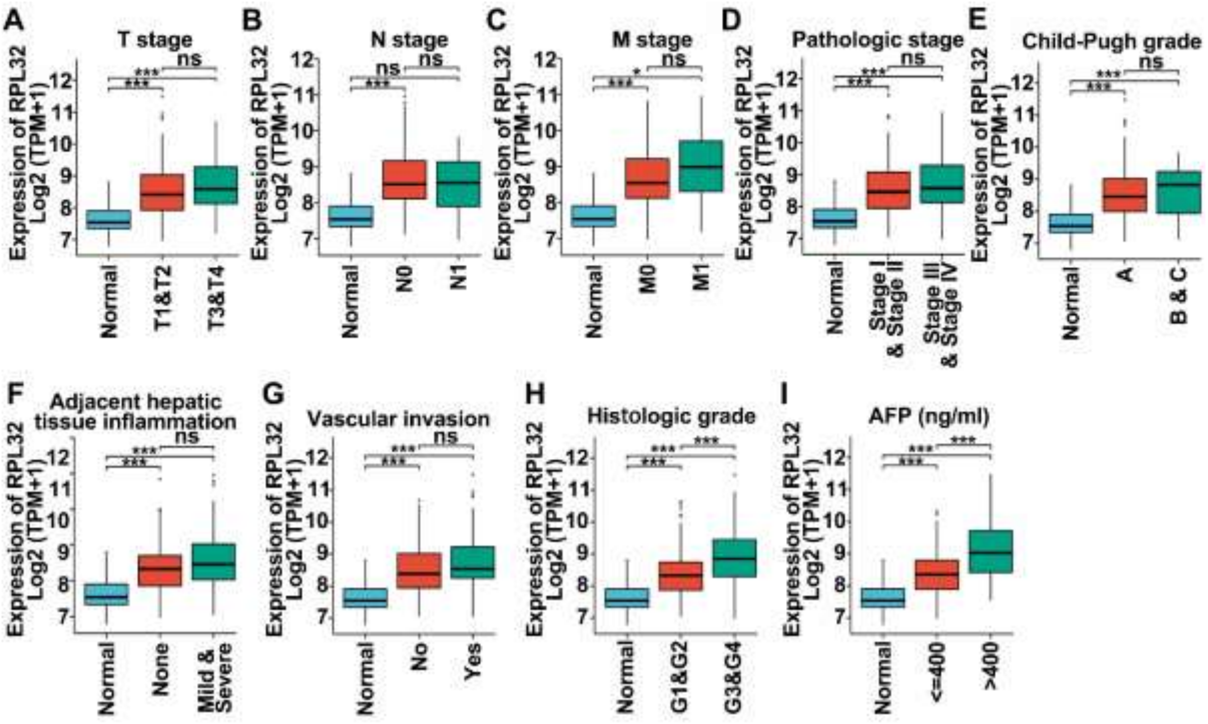

Figure S2. The correlations between RPL32 and clinical stages in the TCGA-LIHC database. (A-I) Differential expression analysis of RPL32 in HCC samples classified by multiple clinical parameters including T stage (A), N stage (B), M stage (C), Pathologic stage (D), Child-Pugh grade (E), Adjacent hepatic tissue inflammation (F), Vascular invasion (G), Histologic stage (H), and AFP concentration (I).

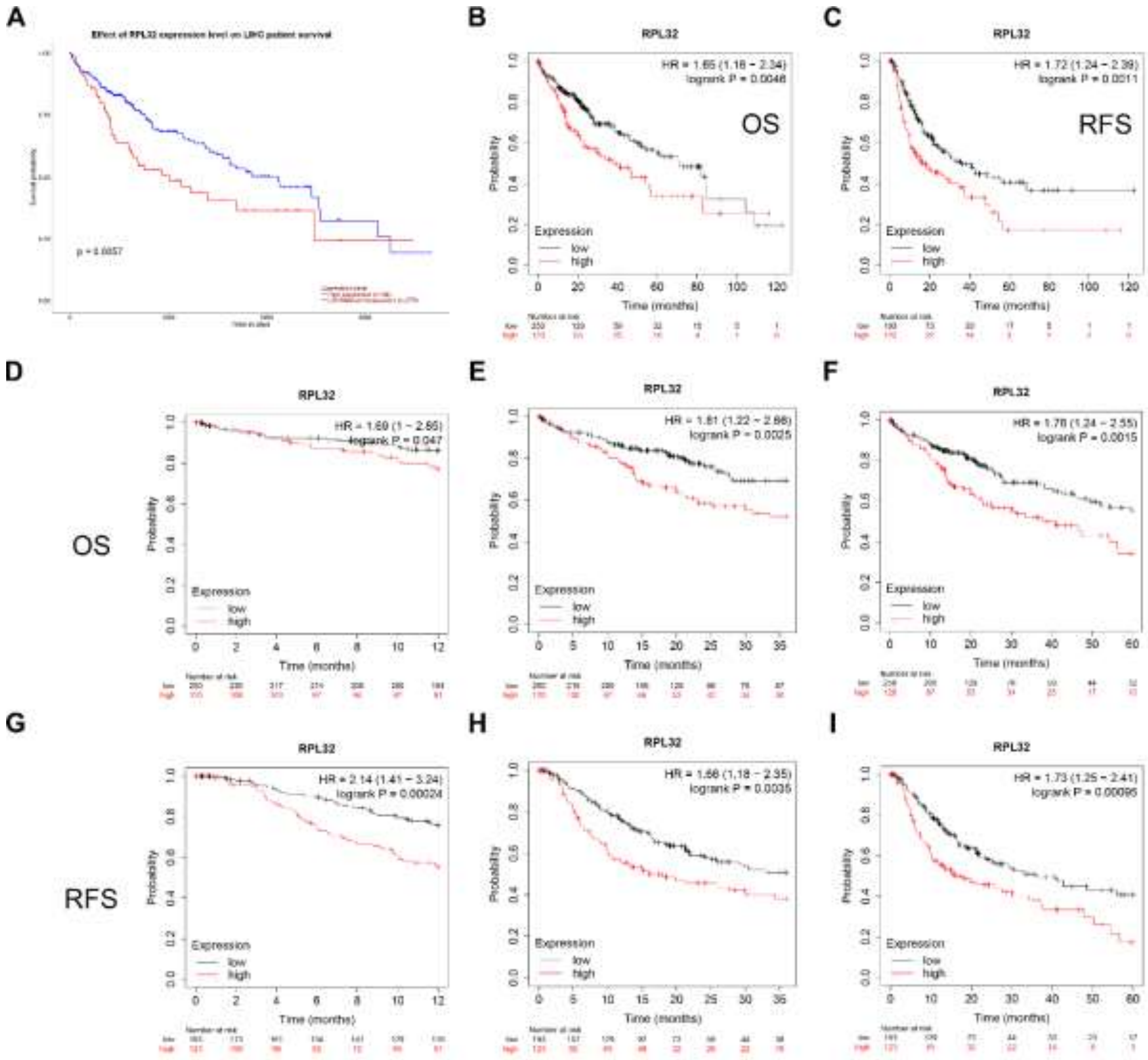

Figure S3. ***RPL32*** **is corelated with the unfavorable prognosis in patients with HCC**

(A) Kaplan-Meier analysis for LIHC patients stratified by *RPL32* expression in UALCAN database. (B, C) Kaplan-Meier analysis (120 months) for HCC patient overall survival (B) and relapse free survival (C) stratified by *RPL32* expression. (D-F) Kaplan-Meier analysis for HCC patient overall survival stratified by *RPL32* expression. The results were represented as 12 months (D), 35 months (E) and 60 months (F), respectively. (G-I) Kaplan-Meier analysis for HCC patient relapse free survival stratified by *RPL32* expression. The results were represented as 12 months (G), 35 months (H) and 60 months (I), respectively. Data of B-I were derived from Kaplan-Meier plotter (https://kmplot.com/analysis/).

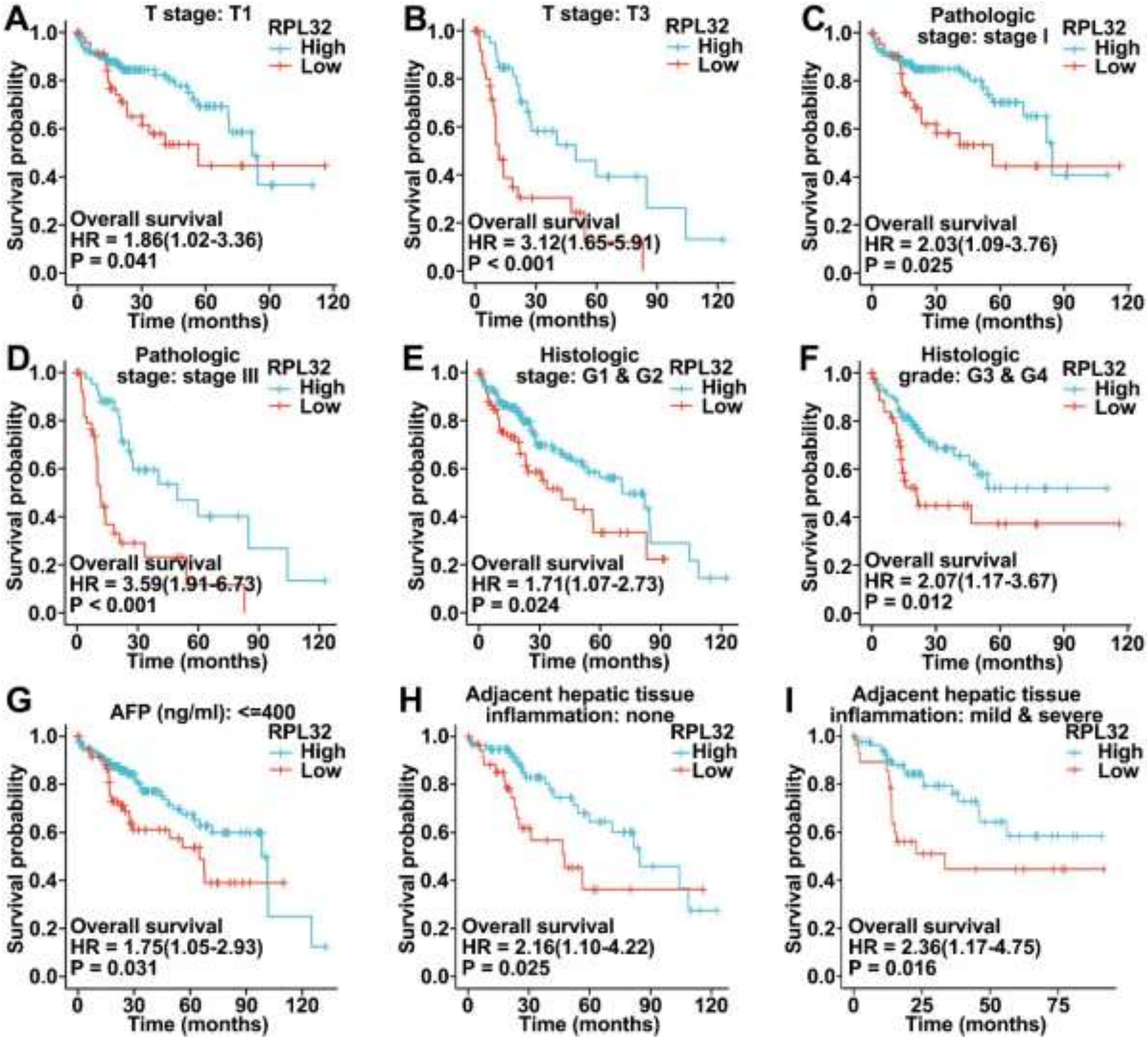

Figure S4. The survival of HCC patients in diverse subgroups.

(A–I) Patient survival analysis of *RPL32* in groups classified by diverse clinical parameters such as T stages (T1, A; T3, B), pathological stages (Stage I, C; Stage III, D), histological grade (G1&G2, E; G3&G4, F), AFP concentration (<=400 ng/mL, G), and adjacent hepatic tissue inflammation (None, H; Mild & Severe, I).

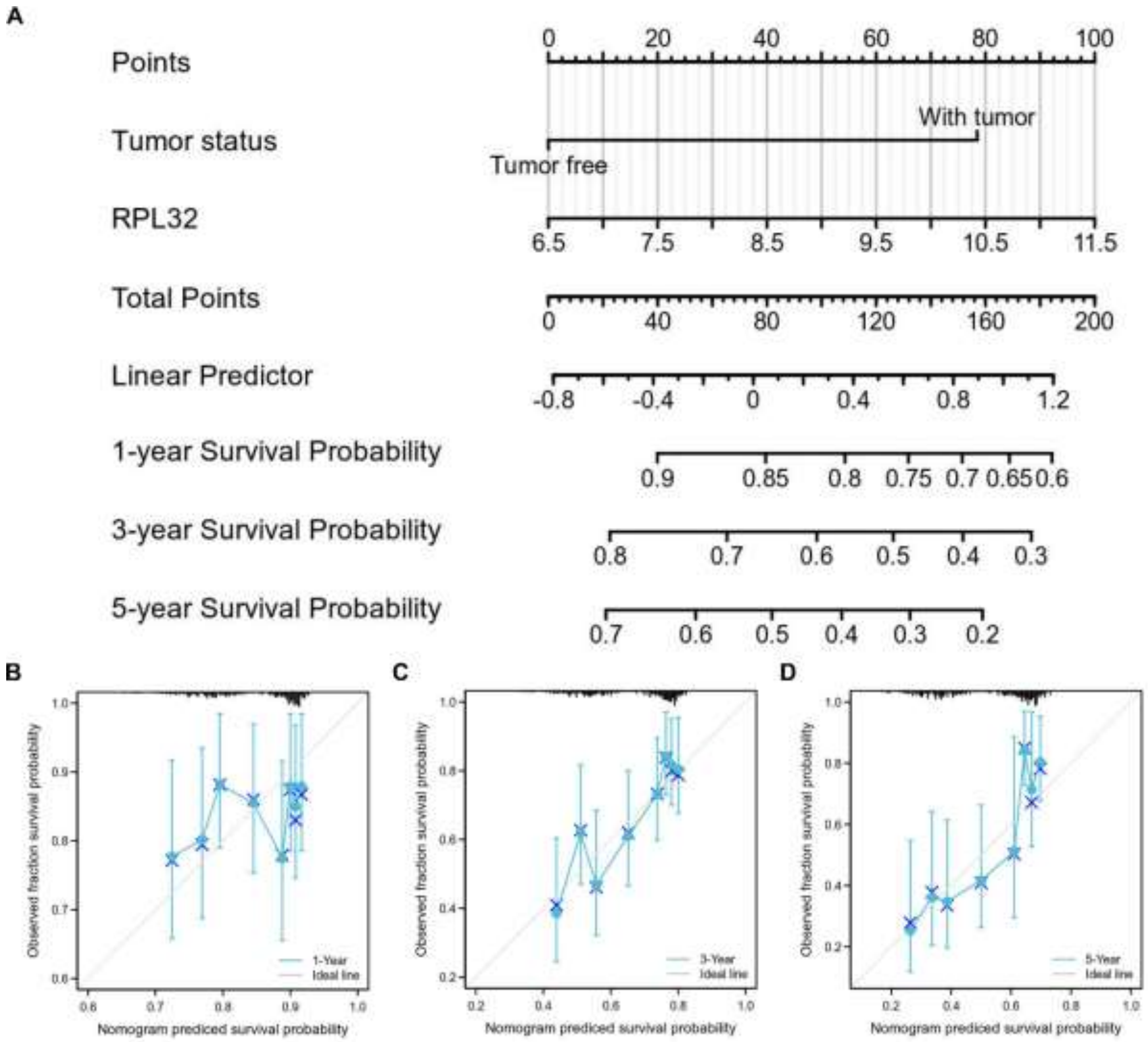

Figure S5. Predict value of *RPL32* for HCC patients

(A) Nomogram for evaluating the risk for a single patient by *RPL32*. (B) Correlation between the actual survival probability of patients and that predicted by the nomogram.

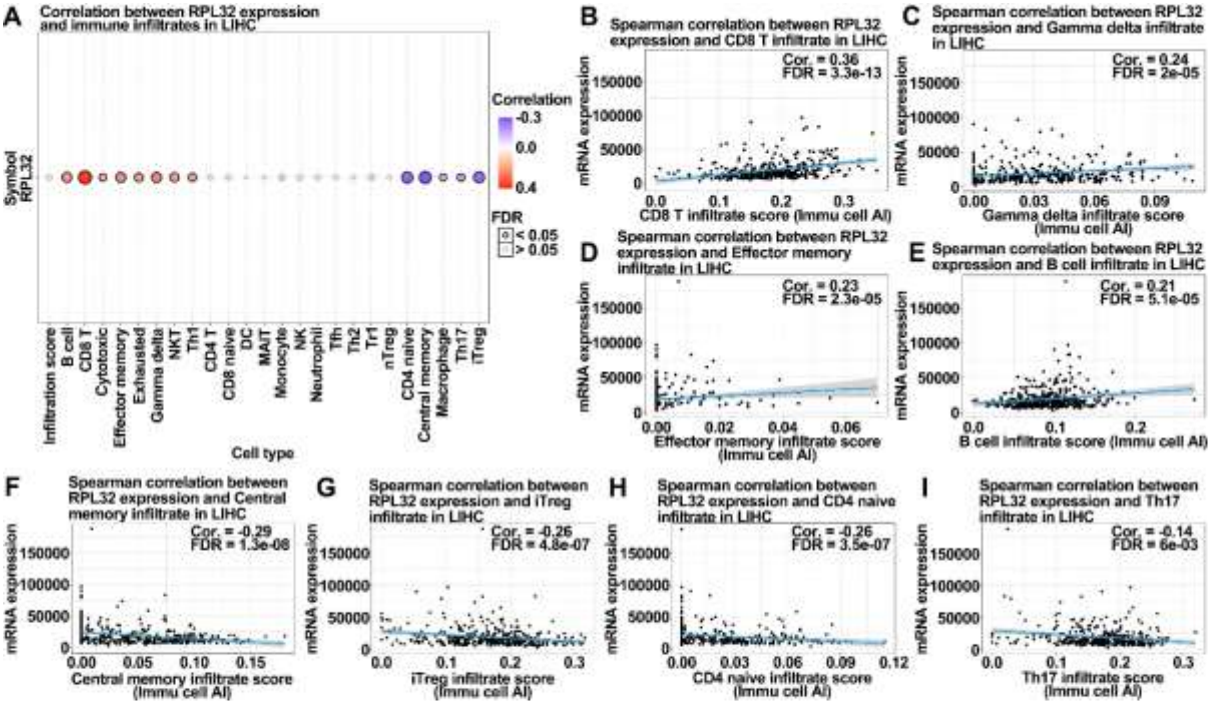

Figure S6. Association between *RPL32* expression and immune cell infiltration

(A) Forest plot showing the correlation between RPL32 expression and diverse types of immune cells. (B-I) Plots showing the association between the levels of RPL32 mRNA and infiltration of various indicated immune cells, including CD8 T (B), gamma delta (C), effector memory (D), B cell (E), central memory (F), iTreg (G), CD4 naive (H), and Th17 (I).

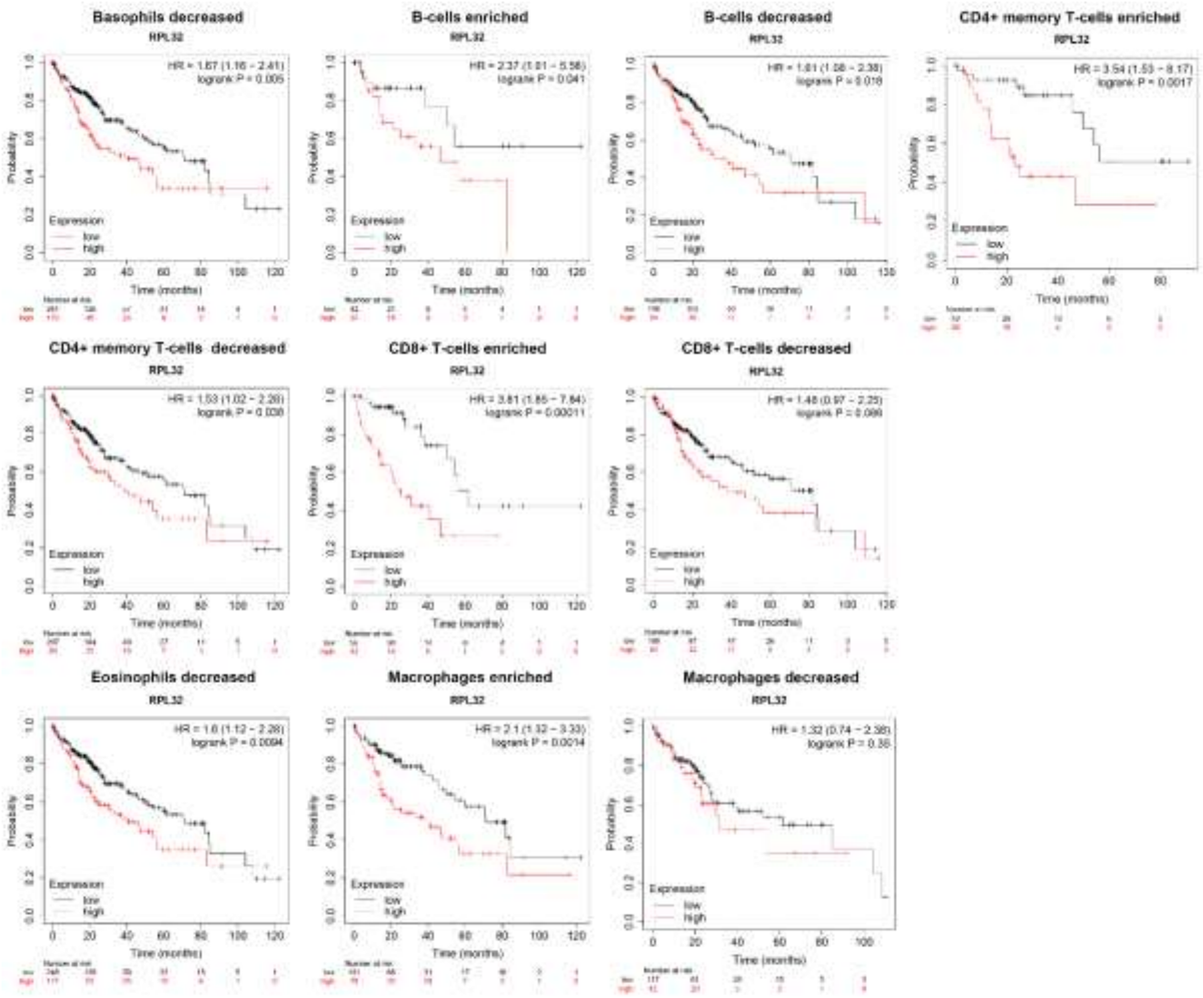

Figure S7. Analysis of *RPL32* expression and patient survival in HCC subtypes divided by immune cell status.

Plots showing the correlations between *RPL32* mRNA levels and HCC patient overall survival in indicated immune subtypes.

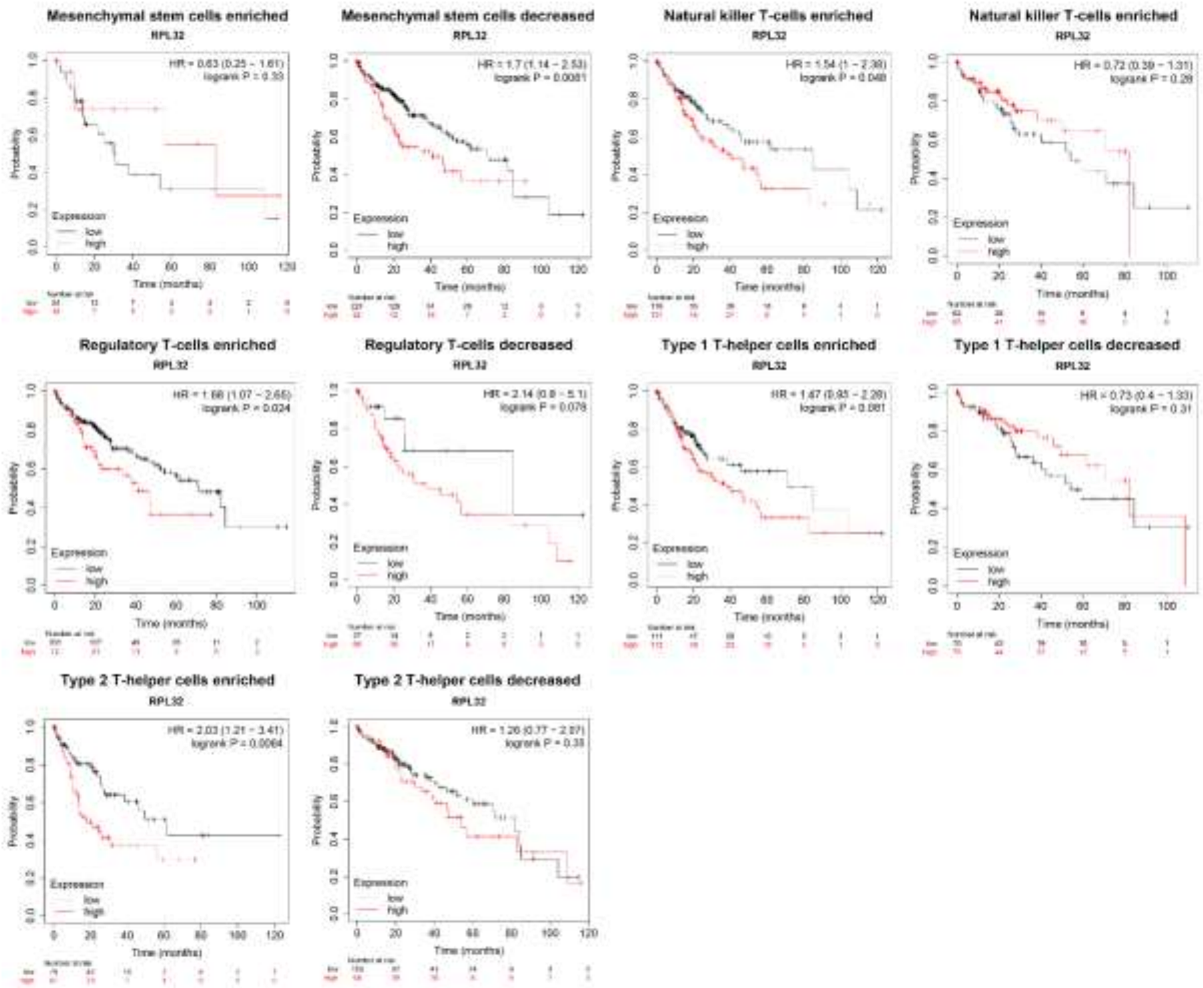

Figure S8. Analysis of *RPL32* expression and patient survival in HCC subtypes divided by immune cell status.

Plots showing the correlations between *RPL32* mRNA levels and HCC patient overall survival in indicated immune subtypes.

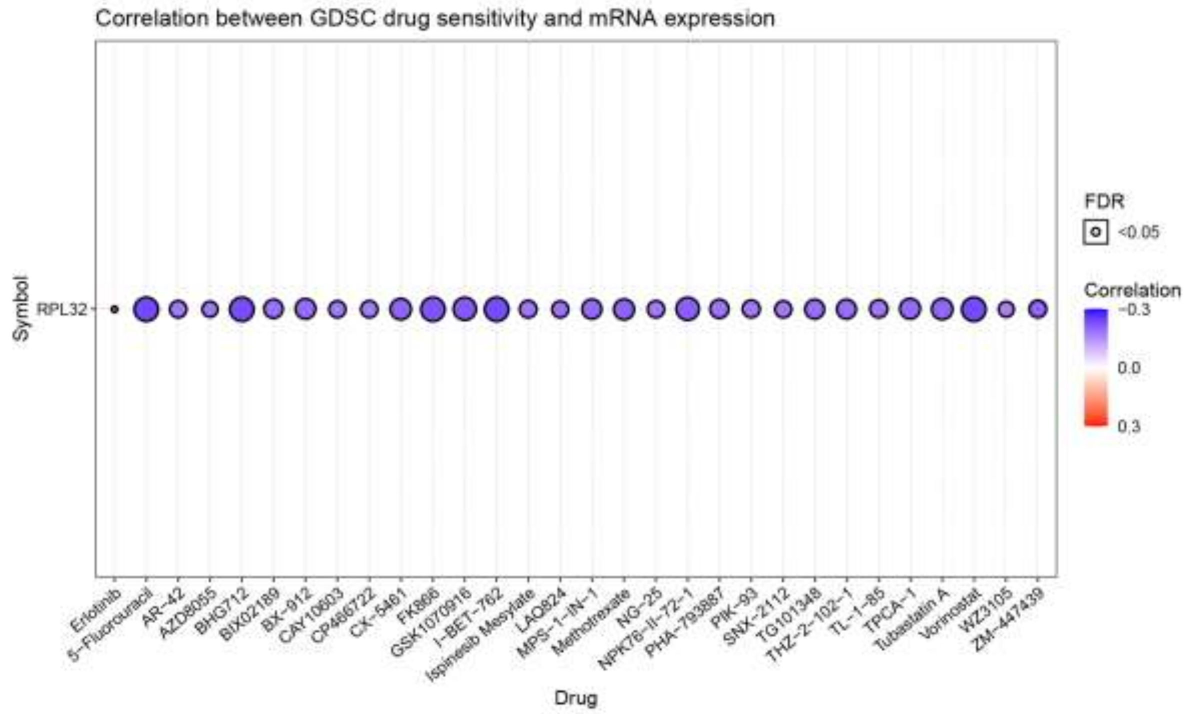

Figure S9. Correlation between *RPL32* expression and drug sensitivity.

Plot showing the associations between *RPL32* mRNA expression and the drug sensitivity from CTRP GDSC database.
